# Promising Concept, Challenging Implementation of a Minimally Invasive Intervention (MINI) to Identify Patients in Their Last Year of Life—A Multi-Methods Feasibility Study at a German University Hospital

**DOI:** 10.3390/healthcare13212784

**Published:** 2025-11-03

**Authors:** Alina Kasdorf, Belinda Werner, Jana Sophie Grimm, Gloria Dust, Steffen T. Simon, Raymond Voltz, Julia Strupp

**Affiliations:** 1 Department of Palliative Medicine, Faculty of Medicine and University Hospital, University of Cologne, 50937 Cologne, Germanysteffen.simon@uk-koeln.de (S.T.S.); raymond.voltz@uk-koeln.de (R.V.); julia.strupp@uk-koeln.de (J.S.); 2 Economics and Social Sciences, Institute of Sociology and Social Psychology (ISS), Faculty of Management, University of Cologne, 50937 Cologne, Germany; 3 Center for Integrated Oncology Aachen Bonn Cologne Duesseldorf (CIO ABCD), Faculty of Medicine and University Hospital, University of Cologne, 50937 Cologne, Germany; 4 Center for Health Services Research, Faculty of Medicine and University Hospital, University of Cologne, 50937 Cologne, Germany

**Keywords:** prognosis, surprise question, palliative care, end-of-life care, terminally ill, decision making, feasibility studies, patient-centered care, hospitals

## Abstract

**Background/Objectives**: Identifying patients with palliative care needs can be challenging in clinical practice. This study reports on the tailoring and evaluation of a minimally invasive intervention (MINI) to support early planning of palliative care in acute hospitals. The MINI includes the Surprise Question (SQ) and the Supportive and Palliative Care Indicators Tool (SPICT^TM^) for health and social care professionals, as well as a patient Question Prompt Sheet. **Methods**: A multi-method intervention study was conducted, including interviews and a pre–post survey of professionals on the development, implementation, and experiences with MINI. Interview data were analyzed inductively and survey data descriptively. **Results**: Data from 44 participants were included. MINI was generally considered acceptable and relevant, particularly the SQ, which prompted reflection among staff. Following the intervention, a significant improvement was observed in the presentation of regional specialist palliative care services for patients, as well as in the identification of psychosocial problems and their discussion with patients and relatives. While physicians reported increased confidence in initiating end-of-life conversations, other hospital staff showed mixed responses. Reported barriers for implementing MINI included limited time, the COVID-19 pandemic, staff strikes, emotional burden, and unclear responsibilities, indicating a low level of commitment. SPICT use was inconsistent, suggesting low integration into workflows. Interprofessional collaboration improved, particularly with external palliative care providers. Sustainability was hindered by a lack of institutional support, ongoing training, and formal routines. **Conclusions**: MINI may have the potential to shift the focus away from purely curative approaches. However, to guarantee success, future studies should ensure better alignment between intervention design, implementation and framework conditions.

## 1. Introduction

The timely identification of patients with palliative care needs remains a major challenge for hospital staff, often leading to late or missed opportunities for palliative care integration [1]. Early palliative care has been shown to improve quality of life, patient satisfaction, and even survival outcomes in various populations [2]. In recent years, Germany has expanded its specialized palliative care services, enabling the provision of such complex and comprehensive care and ensuring the highest possible quality of life for dying patients [3,4]. Although few people in Germany identify the hospital as their preferred place of death, most still die there [5,6]. The hospital plays a significant role as a healthcare setting in the last year of life, with around 9 out of 10 patients having been hospitalized at least once [5]. It is estimated that one in three patients die within a year of hospital admission, with nearly one in two elderly patients dying within this period [7]. Although the hospital plays a crucial role in transitions and care in the last year of life, relatives were the least satisfied with the care received in hospitals [5]. Other studies also showed that there are still discrepancies between palliative care recommendations and the care actually provided in hospitals [8,9].

In fact, recommendations for the care of patients with life-limiting illnesses emphasize the importance of integrating palliative care early on, rather than waiting until the dying phase. Patients themselves also want to be informed about the possibilities of palliative care, but there is a gap in communication with HCPs [10,11]. Palliative care is often only integrated into treatment at a late stage by hospital staff, if at all [9]. Previous studies suggest that a substantial proportion, estimated at around one quarter, of hospitalized patients may have palliative care needs [12,13,14]. In the acute inpatient setting, high workloads, an emphasis on curative treatments and life-sustaining treatments, and the challenge of making accurate prognoses can delay early identification of patients’ palliative care needs by hospital staff [15,16].

Recent evidence evaluating minimally invasive prognostic tools for the identification of palliative needs in hospital settings shows that combining the 12-month Surprise Question (SQ) and the Supportive and Palliative Care Indicators Tool (SPICT^TM^) is a promising method for identifying patients in their last year of life [17,18,19,20]. However, studies have shown that systematic approaches for identifying patients who might benefit from early palliative interventions are still inconsistently applied in hospital settings [21,22]. Building on existing screening tools such as the Surprise Question and SPICT™, this study addresses this gap by developing and evaluating a minimally invasive intervention (MINI) tailored to the acute hospital context.

## 2. Materials and Methods

This study is the second part of the Last Year of Life Study in Cologne (LYOL-C I), hereafter referred to as LYOL-C II. Both parts of the study were conducted in Cologne, Germany. Further information can be found in the published study protocols and related publications [23,24].

### 2.1. Ethical Approval

The study was approved by the Ethics Committee of the Medical Faculty of the University of Cologne (#20-1431), the Staff Council of the University of Cologne, the Equal Opportunities Officers, and the representatives of the severely disabled. This trial is registered in the German Clinical Trials Register (DRKS00022378).

### 2.2. Participants

**Figure 1:**
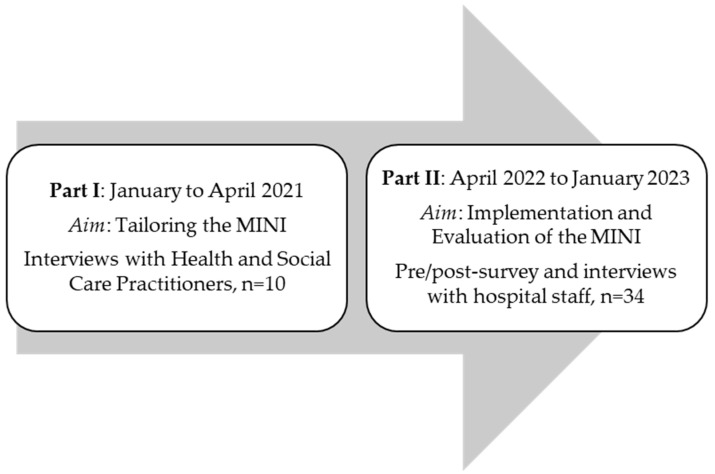
Study program.

A distinction is made between two groups of participants (see Figure 1). The first group consists of those who were interviewed to tailor the intervention to the specific inpatient settings (I). The second group consists of university hospital staff who received the intervention, including a training session and subsequent application, and were later evaluated (Phase II). The participants in part I were health and social care practitioners (HSCPs) providing care for patients in their last year of life. These practitioners were sampled from January to April 2021 and were recruited using a purposive sampling strategy. The participants in part II were hospital staff from two hospital wards at the University Hospital Cologne. These included the gynecology ward, including its outpatient clinic, and an internal medicine ward. Most of the patients were treated for illnesses related to oncology and nephrology. The hospital staff had agreed to attend a Minimally Invasive Intervention (MINI) training. These participants were sampled from April 2022 to January 2023. Information regarding the training was disseminated through the hospital’s newsletter and through announcements by the research team and senior physicians.

All study participants were informed about the study and provided written informed consent.

### 2.3. Intervention

**Part I: Tailoring MINI**

To tailor the MINI to the planned study at a German acute University Hospital, a total of ten HSCP: seven experts (care providers from a predominantly inpatient medical setting) and three patient representatives from Cologne were interviewed. Due to the COVID-19 pandemic, the data collection was shifted to virtual, one-on-one interviews rather than the originally planned group sessions. This format contained in-depth insights into the care situation of patients in their last year of life and highlighted opportunities to improve both the identification of palliative care needs and the provision of care. In addition, determinants relevant to MINI and its implementation were identified to adapt the intervention to the hospital context and to derive targeted implementation strategies. The interviews were also used to tailor the content and design of the newly developed Question Prompt Sheet (QPS) to the needs of patients in the last year of life.

**Part II: Implementing MINI**

Following the validation and adaptation of the intervention to the individual circumstances of the ward and the deliberately selected HSCP, the implementation of the intervention was planned. The conception and design of MINI aimed to facilitate the early identification of patients in their last year of life.

MINI involved the administration of two instruments: the 12-month Surprise Question (SQ: “Would I be surprised if this patient died within the next 12 months?”) and the Supportive Palliative Care Indicator Tool (SPICT-DE^TM^) for HSCP [25]. SPICT-DE^TM^ provides a structured set of general and disease-specific indicators to identify patients with deteriorating health who may need palliative care. A QPS titled “Conversation Guide for Your Healthcare” was also provided, intended for both patients and their care partners [23]. Both SQ and SPICT-DE^TM^ were available and validated in German [25,26]. The intervention study comprised two main components: HSCP-related and patient-related. HSCP-related components were aimed to enhance HSCPs’ awareness of palliative needs by conducting MINI-training designed to equip them with the necessary skills to utilize the intervention. Patient-related components were to emphasize improving patient outcomes in comparison to the control group. As this study focuses on the HSCP-side intervention, the use of QPS is not explicitly evaluated herein (for further details regarding patient outcomes of MINI, see Grimm et al., 2025 [24]).

The intervention comprised providing diverse resources, such as updated posters, flyers, and an (online) MINI-training course for members of the ward staff, including physicians, nurses, and case managers. Key stages of implementation involve distributing materials at strategic points during care (e.g., after initial treatment or before physician consultations) and ensuring proper handovers of responsibility between staff, including healthcare providers and the study teams. The MINI training emphasized fundamental principles of palliative care, the importance of early integration, and effective communication strategies for discussing terminal diagnoses with patients and their families, utilizing the SPIKES communication model (Setting, Perception, Invitation, Knowledge, Emotions with Empathy and Strategy) [27], delivered by an experienced palliative care physician, among others. Following the training program, staff were guided to apply the SQ alongside the SPICT-DE™ to identify patients in their last year of life. Each training, including the educational lecture, presentation of MINI, and case discussions, lasted approximately 60–90 min. A survey questionnaire was administered prior to and following the MINI training (pre–post survey).

### 2.4. Instruments

#### 2.4.1. Interview Guide

Qualitative data were assessed for both groups of participants.

All interviews were audio recorded and transcribed verbatim. The STROBE checklist was used to guarantee thorough and transparent reporting of all critical elements of the quantitative investigation [28], while the COREQ Checklist (Consolidated criteria for Reporting Qualitative research) was used to report the qualitative data [29].

Tailoring interviews with HSCP n = 10: Interviews included the following questions: Presentation/discussion of MINI: In order to facilitate effective realization and implementation of the MINI, what is required? What design suggestions do you have? Does MINI meet the needs? How would you assess the feasibility of implementation? How do you rate the content of MINI? What additional information would you add? Is more disease-specific information/criteria considered important? In what form and how would you present this tool on the ward? Who should use the tool? How? And when? What do you think should be considered in the day-to-day use of the intervention? How can the long-term use of the tool be maintained? With regard to the implementation of the intervention, which barriers do you consider to be of significance, in order to be able to tailor our intervention as closely as possible to the requirements?

Evaluation interviews with hospital staff members n = 10: Interviews included the following questions: What does ‘palliative care’ mean to you? Have you used the intervention? Did applying the intervention change your perspective on palliative care needs? What could we have done better? Which patient groups do you use MINI for? How would you rate the intervention overall, i.e., the benefits of the intervention?

Purposive sampling was utilized to select participants for interviews, with the criteria including attendance at the training and a sufficient level of motivation to discuss their experience with MINI. The interview guide was presented and discussed in a qualitative research workshop with the research group in September 2022. A positive vote from the staff council was obtained in December 2022. The number of interviews conducted depended on reaching thematic saturation regarding the research question. A total of five interviews per hospital unit were planned, involving staff from different professional groups engaged in the intervention’s implementation. Individual interviews were conducted due to scheduling constraints across professional groups with survey participants. The interviews were planned to take place at the University Hospital. The data collection took place from December 2022 to August 2023. The evaluation interviews were conducted by two female research associates of the Cologne Research and Development Network (CoRe-Net) study group members with experience in conducting qualitative research. It is noteworthy that the interviewer was not the presenter of the MINI training.

#### 2.4.2. Questionnaire

To evaluate the MINI, hospital staff members underwent a pre–post survey by using a self-developed questionnaire consisting of 30 (pre-) and 36 (post-) items. The questionnaire was available both online and on paper, with the online survey conducted via the LimeSurvey platform at the University of Cologne. The data was collected between April 2022 and August 2023, as shown in Figure 1, via an online survey. The questionnaire was based on the German National S3 guideline for palliative care (providing recommendations for structured hospital conversations addressing patients’ physical, emotional, social, and spiritual needs, life goals, and end-of-life care) and findings from the previous study [30,31]. It collected demographic data and assessed self-reported competencies in areas such as identifying patients in their last year of life and addressing patients’ wishes and needs through communication with patients and involved caregivers. The response format for the self-developed items consisted of a 4-point Likert scale with the options: ‘agree’, ‘somewhat agree’, ‘somewhat disagree’, and ‘disagree’, as well as a ‘no response’ option. The questionnaire also included open-ended questions allowing participants to elaborate on their answers and provide further comments.

To assess palliative self-efficacy, the Palliative Competence Test for Physicians (Palliative Kompetenztest (PKT), a validated 52-item tool, was used [32]. The self-efficacy scale of PKT was adapted for use in an acute care setting and across different professional groups by removing 8 of the 10 original items. As the PKT is designed for formative rather than diagnostic purposes, no cut-off values were defined. Permission to use the adapted version was obtained from the author. Some questions about communication on death and dying were based on a doctoral thesis by Hauck (2007), with content adapted to the study’s aims and the response format standardized to align with the rest of the questionnaire [33].

To ensure comprehensibility and practicality, the entire questionnaire was initially presented and discussed in a research workshop with staff from the Department of Palliative Medicine at the University Hospital of Cologne. A revised version was pre-tested using the think-aloud technique, resulting in changes to wording and response format.

Data collection was pseudonymized. Participants created a personal code that could only be traced back to them, to allow them to exclude their data if they wished, as well as to analyze individual differences in the pre–post survey.

### 2.5. Analyses

The qualitative data were analyzed by following the principles of qualitative content analysis [34]. The analysis followed Mayring’s structured approach to qualitative content analysis, consisting of summarizing, explicating, and structuring the material to derive inductive categories that reflect the essential content in a systematic and transparent manner. A common deductive framework was applied, reflecting the research questions of the evaluation. The Consolidated Framework for Implementation Research (CFIR) was used to analyze contextual determinants during the tailoring phase [35]. Proctor’s and colleagues’ implementation outcomes framework guided the evaluation of the implementation phase [36]. The subcategories of acceptability, appropriateness, feasibility, fidelity, and sustainability were derived deductively from Proctor’s and colleagues’ framework and categorized using the available interview material. Although the overall dataset also included inductively coded material, only the deductive categories were used for this publication to ensure theoretical consistency with the implementation focus. A detailed codebook was developed and applied consistently across all interviews to ensure transparency and reliability. Two researchers (AK and JS) had regular coder meetings to ensure the precise classification of categories. The sample size was considered sufficient based on the concept of information power [37], as the interviews provided rich, relevant, and specific information to address the research questions.

Quantitative data was analyzed descriptively using IBM SPSS Statistics 28. Responses “Don’t know” were treated as a missing value; for other responses, the answers have been summarized in a dichotomous format (“Agree” including the responses “agree” and “somewhat agree”, “disagree” including the responses “somewhat disagree” and “disagree”). Pre–post comparisons were conducted using the Wilcoxon signed-rank test. Statistical significance was assessed using unadjusted *p*-values, with an alpha level of 0.05. No correction for multiple comparisons (e.g., Bonferroni) was applied, as the analyses were exploratory in nature.

Due to the limited sample size, inferential testing (Wilcoxon signed-rank test) was conducted in an exploratory manner, and results are interpreted descriptively without claims of generalizability.

The results are presented according to Proctor et al.’s implementation outcome categories (acceptability, adoption, appropriateness, feasibility, fidelity, implementation cost, penetration and sustainability), reflecting the deductive coding of the qualitative data [30]. To provide a structured overview of the implementation experience, all data were translated into numeric scores (0 = not achieved, 1 = partially achieved, 2 = fully achieved; n/a = not applicable). The scoring was developed inductively based on the qualitative interview data and the corresponding quantitative survey trends, following researcher consensus. Each score represents the extent to which an implementation outcome, as defined by Proctor and colleagues’ framework, was evident across data sources. For example, partially achieved indicates that the respective implementation criterion (e.g., feasibility or acceptability) was supported by some, but not all, qualitative statements or quantitative indicators.

## 3. Results

**Part I: Tailoring MINI**

The interviews were conducted to explore possible barriers to the implementation and necessary tailoring of the intervention and implementation strategies with regard to the hospital setting and resources. We assumed that a successful implementation of the SQ and SPICT intervention requires a well-planned combination of clear training, regular reminders, intensive interdisciplinary collaboration, and a clearly structured process. Employee motivation and open, transparent communication within the team were identified as key factors in this process.

The tailoring of MINI focused on addressing multiple categories to enhance accessibility and relevance for hospital staff. Tailored strategies included the continuous availability and presence of study staff, as well as regular updates of materials. The implementation strategy was designed to be flexible and patient-centered, ensuring that the intervention could be adapted to the needs of hospital staff and seamlessly integrated into their workflows.

Key barriers for a successful intervention were identified in the areas of content, language, format, and implementation strategies. The content of MINI was tailored to include diverse examples, clear language, and updated contact information. The layout was designed for readability, with appropriate font sizes and high contrast to improve accessibility. Long-term accessibility was ensured through regular updates and the dissemination of MINI through various channels, including digital formats and printed copies in staff areas.

**Part II: Implementing MINI**

### 3.1. Study Population

**Figure 2:**
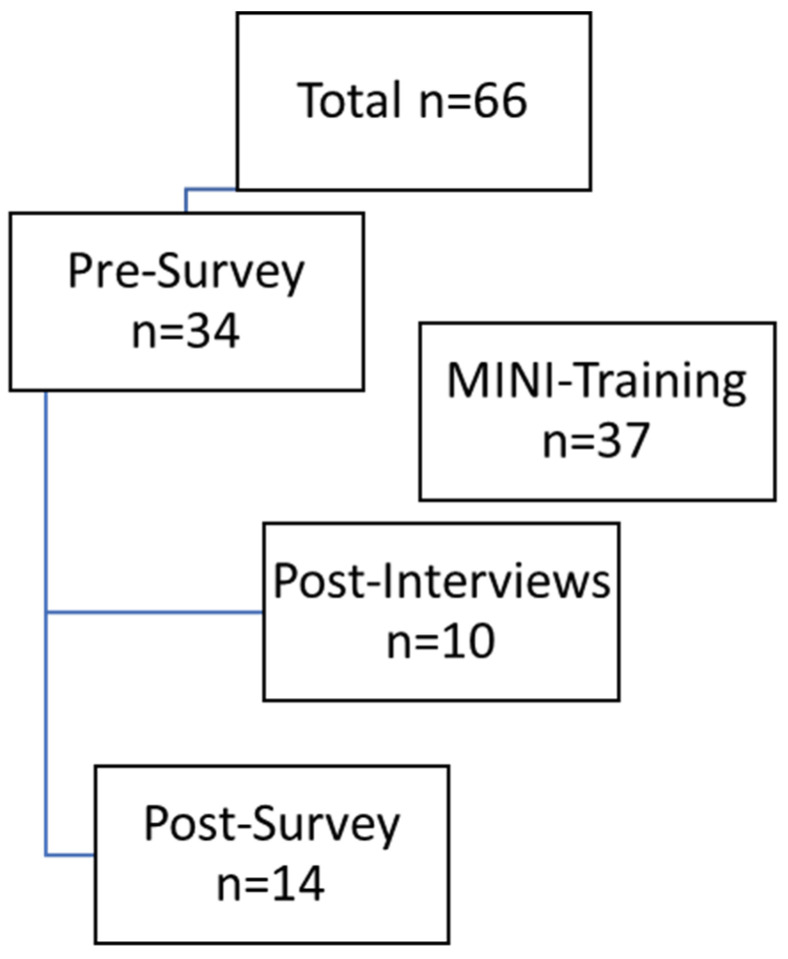
Data generation process.

In total, there were 66 HSCPs, including 20 doctors, 40 nurses, and six other professionals (study nurse, case manager), as shown in Figure 2. The pre-survey was completed by 34 people, representing 51.5% of those invited. At baseline (t0), the sample size was already smaller (n = 34) than expected (n = 66), and by the follow-up (t1), a substantial number of participants had dropped out (n = 14). Thus, only 41.2% of participants from the pre-survey completed the post-survey.

The lower number of participants was due to some employees switching to other wards, leaving the organization, or being unavailable by email or telephone. Employees received an incentive of €25 in the form of a voucher for participating in both surveys. Five (35.7%) of the 14 participants who completed the post-survey indicated that they had moved to a different ward, which made it difficult to assess how the intervention had worked. In conclusion, a total of ten interviews were conducted with participants following their participation in the MINI training program. Six HSCPs worked on the non-oncology ward and four on the oncology ward. Table 1 presents the sociodemographic profile of the study participants.

### 3.2. Evaluating MINI

The evaluation of the MINI reveals substantial variation across implementation dimensions, highlighting both conceptual potential and operational limitations.

#### 3.2.1. Acceptability

 **Definition.** *How well is MINI accepted by the involved HSCP?*

Overall, the MINI was well accepted, although experiences varied across professional groups and intervention components (Table 2). In the quantitative survey, a total of 90.0% of respondents found the SQ helpful, with the satisfaction rate among physicians at 100.0%. In qualitative interviews, however, views were more ambivalent. While some participants valued the SQ as a stimulus for reflection and collegial exchange, others described it as emotionally demanding, particularly when confronted with the tension between clinical responsibility and the acknowledgment of dying:

*“And then you have to deal with your inner level, so to speak, and say: ‘Well, no, so if I really look at it very closely and soberly, then it’s actually like that, yes.”*

(Interview 10)

Among physicians, the supplementary tools (SPICT, QPS) were not seen as helpful at all (0% positive feedback). Overall, only 14.3% of participants found them useful. Barriers included limited applicability in routine workflows and perceived complexity, as reported by the respondents:

“*It’s good to read up on, I think, but in parts it’s a bit much* […]”

(Interview 05)

Despite such reservations, overall satisfaction with the intervention was high (91.7%). A majority (71.4%) considered the time investment worthwhile, particularly physicians (83.4%). The intervention also had an influence on interprofessional communication. One interviewee emphasized the SQ’s potential to initiate conversations across professional hierarchies.

“*But the fact is that deliberately posing the question definitely draws attention to the topic, which is a good thing, it enables people to engage with seriously ill people or with the illness situation and that you can then also discuss it with younger colleagues, for example, and that you can then pass it on as CULTURE. So in that respect, the intervention was good. It woke things up. And now it would be good if we could stabilize it, so to speak. I would say that. So the question is definitely important, and the issue is right. We just need to be able to anchor it more firmly, yes*.”

(Interview 10)

Quantitative findings on interprofessional exchange showed mixed trends. Communication about patient needs within the ward team declined overall (from 58.8% to 50.0%), mainly due to a marked drop in physician-reported rates (from 61.5% to 33.3%). In contrast, non-medical staff reported stable or improved communication (from 57.1% to 66.7%). Notably, collaboration with external providers increased across nearly all domains: communication with specialists rose from 58.8% to 81.8%, with general practitioners from 22.6% to 36.4%, and with specialized outpatient palliative care from 53.3% to 66.7%. Contact with palliative care services and relatives remained consistently high (82.4% to 91.7% and 91.2% to 92.3%, respectively).

In terms of patient-centered care, physicians reported increased, though not statistically significant, engagement in key end-of-life conversations: regarding advance directives, power of attorney, upcoming decisions (84.6% to 100%, respectively), and preferred place of death (from 53.8% to 83.3%). Among non-medical staff, these changes were less pronounced.

#### 3.2.2. Adoption

 **Definition.** *To what extent is MINI implemented in practice?*

The adoption of the MINI into routine clinical practice was heterogeneous (Table 3).

Although general willingness to engage was high particularly among physicians, its consistent implementation remained limited. Competing clinical priorities often leads to de-prioritization, especially among nursing staff and assistant physicians. While the intervention tools were available and known, they were frequently viewed as ancillary to core clinical tasks. A lack of defined follow-up structures and unclear responsibilities further impeded implementation:

“*That’s not easy to do more often. We have a meeting with the physicians here in the morning, we’ll discuss it then. But it’s only being touched on lightly. So it’s not satisfactory. […] They’re there, everyone knows them […] But anything additional is massively cut back right now. That really has to come from the top.*”

(Interview 02)

Self-assessments of palliative care competencies showed improvement, particularly in psychosocial domains (Table 4). Confidence in recognizing and addressing psychosocial concerns increased significantly (from 73.5% to 84.6%, *p* = 0.026), with the largest gains observed among non-medical staff (from 57.1% to 87.5%). Familiarity with regional specialized palliative care services improved (from 38.2% to 50.0%, *p* = 0.020), as did the ability to initiate referrals (from 57.6% to 69.2%). All participants reported post-intervention confidence in identifying complex needs at the end of life (100%). Other indicators demonstrated less consistent patterns. Among physicians, confidence in presenting local palliative care options declined slightly (from 38.5% to 33.3%), as did confidence in advising on advance directives (from 61.5% to 50.0%). In contrast, other staff reported gains, for instance, confidence in discussing living wills rose from 33.3% to 66.7%.

Participants also reported improvements in the identification and management of patients in the last year of life. Knowledge of relevant criteria for patients being in their last year of life increased from 82.4% to 92.9%, and confidence in recognizing such patients rose from 73.5% to 84.6%. While perceived ability to provide care improved modestly (from 72.7% to 78.6%), more pronounced gains were observed in participants’ confidence in communicating life-limiting diagnoses (from 58.8% to 75.0%). These improvements were most pronounced among physicians, although other professional groups also reported positive changes.

#### 3.2.3. Appropriateness

 **Definition.** *How suitable is MINI for the target group and the context?*

The appropriateness of the MINI appears to be highly context dependent (Table 5). 

Interview data highlighted that successful implementation relied strongly on who initiated the intervention, when, and in which clinical setting. A sensitive, individualized approach was considered crucial, particularly by nursing staff, who often maintain closer emotional proximity to patients. At the same time, physicians were perceived to hold the greatest authority, especially regarding prognostic or conclusive statements, thereby reinforcing existing role hierarchies:

“*[…] There are emotional hierarchies: When a doctor says something, it’s different from when a nursing staff or case manager does.*”

(Interview 02)

Application of the SQ varied across departments and was seen as less useful in certain contexts. For example, in nephrology or chronic care settings, the SQ was often perceived as insufficiently specific, given the high proportion of patients with poor but fluctuating prognoses. Even when criteria were met, the subsequent steps toward care planning were often lacking, indicating a disconnect between assessment and actionable change.

Interviewees emphasized that the relevance of the MINI was closely tied to the disease stage and the patient’s willingness or ability to engage in prognostic conversations. Some patients avoided such discussions, while others were too emotionally or physically compromised to participate. Although MINI occasionally prompted earlier reflection, its introduction frequently occurred too late. The hospital environment, described as reactive and crisis-driven, was perceived as suboptimal for adopting such interventions by the respondents, especially when compared to primary care, where longer-term, continuous relationships could enable earlier engagement.

Relational dynamics were also identified as key to acceptance. Nursing staff were viewed as more emotionally attuned facilitators, while physicians were seen as more credible in delivering prognostic information. This dual perception created a structural tension: the effectiveness of communication was shaped not only by what was said, but also by who delivered the message.

Despite these challenges, the intervention may have contributed to more favorable perceptions. Over half of the respondents (57.1%) reported that MINI had changed their view of palliative care needs, mainly through improved interdisciplinary communication and earlier recognition of relevant issues. In parallel, the proportion of staff who considered the hospital an appropriate setting for end-of-life discussions increased (from 78.8% to 92.9%). Furthermore, the belief that patients struggle to express fears about dying to hospital physicians declined (from 91.2% to 71.4%), indicating a potential shift in communication culture.

#### 3.2.4. Feasibility

 **Definition.** *How feasible is the implementation of MINI?*

The feasibility of the MINI varied considerably across its individual components (Table 6). 

The SQ was still rated most positively: most respondents considered it comprehensible (90.0%), applicable (90.0%), and manageable in terms of time (90.0%). In contrast, the SPICT and, particularly, the QPS received substantially lower ratings. Only one-third of respondents were satisfied with the time investment required for SPICT (33.3%) and 40.0% for QPS. These instruments were also rated lower in terms of being comprehensible (57.1% and 66.7%) and applicable (42.9% and 50.0%).

A major barrier to implementation lies in the structural and organizational context, as reported by respondents. Shift work, staff shortages, and limited time windows hinder continuous and interprofessional coordinated application. Coordinating between nursing staff, physicians, and case management was frequently described as demanding. Although there was repeated interest in regular, structured meetings, the current implementation remains largely ad hoc and dependent on individual commitment, arguing against sustainable routine integration.

A particularly critical issue is the discrepancy between the perceived theoretical value of the intervention and its practical applicability. While the MINI is considered conceptually suitable, its application often fails due to the realities of everyday clinical practice. Without structural adjustments, such as defined time slots, clear responsibilities, and simplified materials, the intervention remains difficult to scale, as reported by the participants.

#### 3.2.5. Fidelity

 **Definition.** *To what extent is MINI being implemented in accordance with the original specifications?*

Several components, in particular SPICT, were modified in the application by being used selectively or omitted entirely (Table 7). 

These adjustments were often made to navigate profession-specific barriers or to enhance practical applicability in day-to-day routines. Qualitative data suggests that the intervention was primarily used as a cognitive prompt rather than as a standardized protocol. Especially among experienced nursing staff, the content was reportedly internalized to such a degree that formal application became less relevant in practice. One physician stated:

“*So we can’t avoid asking ourselves the question: palliative care or not? And I’m happy to take this note with me again. […] As I said, I don’t think I’ll go into the patients’ […] ask myself point by point: Does this apply or not*?”

(Interview 03)

These findings are supported by quantitative data: while the SQ was used regularly by 75.0% of respondents, the use of SPICT remained maximally inconsistent, with 0.0% agreement in this item. This variation in adherence underscores a central challenge: the intervention was not applied as an integrated package, but rather in a fragmented and context-dependent manner.

External factors further contributed to limited fidelity. The COVID-19 pandemic significantly disrupted study procedures: hygiene restrictions, visitation bans, staff shortages, and the loss of key personnel led to gaps in implementation, evaluation, and training. In addition, the multi-month nursing strike (April–July 2022) had substantial consequences. Wards participating in the study were temporarily closed, untrained staff had to take over, and systematic application of the MINI became virtually impossible. As a result, implementation was highly fragmented and largely dependent on the initiative of individual staff members and the research team.

#### 3.2.6. Implementation Costs

 **Definition.** *What financial resources are required for implementation?*

Financial resources were hardly discussed (Table 8). 

However, it became clear that the introduction and maintenance of the intervention require human resources, in particular time and coordination. Establishment as a standard process was only considered realistic if continuous personnel resources, e.g., through trained specialists or regular further training measures, could be provided. With regard to indirect cost efficiency, the survey data shows that the average time required for patients who are presumably in the last year of life has changed from 7.36 min (SD 8.5) to 13.07 min (SD 14.9) for respondents as a result of the intervention. Accordingly, for 23.1% of respondents, the time required has increased, and for 7.7% it has been considerably extended. 71.4% considered the intervention to have been worth the time invested, while 28.6% rated it as neutral.

#### 3.2.7. Penetration

 **Definition.** *How widespread is the MINI within the target population?*

The visibility and reach of the MINI within the target population were heterogeneous and partially low (Table 9). 

The intervention’s penetration within the clinical setting appears to be limited to specific departments and patient groups (e.g., predominantly tumor patients). Despite some integration in routine practice, broader dissemination is hampered by logistical issues and varying levels of staff engagement. One interviewee criticized the non-uniform application of the intervention, which was developed on the basis of HSCP input. This selective penetration limits the impact on a broad level. It was found that the intervention was primarily implemented by senior physicians, potentially excluding other professional groups from active use. Change in responsibilities, the general workload and hierarchical structures in everyday hospital life also made it difficult to implement the intervention. Many HSCPs who were introduced to the intervention and were supposed to suggest patients for the study team by use of the MINI were difficult to reach, left the participating wards after some time or stated that they did not have time to implement MINI in addition to patient care.

#### 3.2.8. Sustainability

 **Definition.** *To what extent can the MINI be maintained in the long term?*

A number of prerequisites were identified as being necessary for the long-term implementation of the intervention (Table 10). 

The implementation of recurrent training sessions, designated meeting times within the team, and the utilization of visible reminder formats, including posters and displays, were identified as conducive to effective team functioning. Materials such as posters and SPICT displays were present on the ward; their perception was strongly influenced by individual attention focus and context (e.g., proximity to the workstation). The personal presence of the research team was described as crucial for recall and use.

At the same time, it became clear that the hospital requires structural routines to ensure widespread adoption of new instruments. Respondents generally indicated a high level of willingness to retain elements of the intervention but emphasized the difference between individual motivation and systematic implementation.

Continued interest in using the MINI was high, reported by 75.0% of all respondents (83.3% among physicians; 66.7% among other staff).

MINI appeared to initiate reflective processes within some teams, which occasionally extended beyond the study period. In selected settings, elements of the intervention were embedded into routine ward rounds and interdisciplinary case discussions, facilitating more differentiated and collaborative care planning.

## 4. Discussion

This study describes the evaluation of a low-threshold intervention aimed at supporting the early initiation of palliative care planning in acute hospital settings. The findings of the present study suggest that MINI may provide a minimally invasive stimulus for reflection on the ethos of curative care. However, while the SQ is regarded as a pragmatic and accessible catalyst for introspection, it has been demonstrated that SPICT and QPS are employed only sporadically as a preliminary guideline for the initial implementation. An analysis of the intervention’s outcomes at the patient level, as reported by Grimm et al. (2025), suggests that MINI may influence aspects of the curative mindset [24], though results from the current study remain preliminary.

The intervention was generally well-received. MINI may foster interdisciplinary discourse and could support engagement with the subject of serious illness, dying, and death. Physicians showed indications of increased knowledge and self-awareness regarding the identification and management of patients in their last year of life compared to other medical staff. Some improvements were observed in recognizing relevant criteria and caring for these patients. The involvement of trained personnel may support patient communication and inter-team collaboration.

Satisfaction with the intervention is also reflected in its future prospects, with 80% of physicians expressing a desire to continue with the intervention. Physicians reported some increases in confidence in utilizing the intervention and in addressing palliative care aspects, including psychosocial concerns.

As indicated in previous studies on the SQ [38,39], its function as a low-threshold trigger for prognostic considerations has been emphasized. The results of our study lend further support to this finding. However, it is crucial to emphasize that the prognostic quality of MINI was not a subject of intervention, and no measurements were taken to ascertain the accuracy of the identification of a last year of life patient at any point in time. Internationally, the combination of the SQ with the Supportive and SPICT has received empirical support as a feasible and clinically valuable approach to identifying patients in their last year of life [20,40]. Research conducted in the domains of acute care and oncology has demonstrated the capacity for seamless integration of these instruments into established clinical workflows [41,42]. The extant results indicate that the service providers benefited exclusively from the use of the SQ. SPICT was utilized to a negligible extent, despite its adaptation and validation for the German healthcare system [25]. However, it can be hypothesized that the outcomes would have been even more favorable if SPICT had been digitally integrated into the hospital information system, as was the case in the study by Van Wijmen et al. [18].

MINI can trigger ambivalent reactions among medical staff, especially if it is understood as an implicit request to limit healing efforts. This is consistent with the findings that prognostic tools can trigger emotional discomfort and ethical uncertainty in clinicians, potentially leading to moral distress. These are recognized barriers to the adoption of prognostic tools as they can cause moral discomfort in healthcare professionals [43,44,45]. Our findings confirm this and highlight the emotional challenges faced by staff when engaging in end-of-life conversations.

International findings also show that the implementation of such interventions is associated with specific challenges. These barriers include lack of confidence in dealing with prognostic assessments, lack of time, cultural barriers, “rescue culture” in the hospital and concerns about the potential impact on the doctor-patient relationship [46,47,48]. At the same time, a dilemma arises: the structural conditions in inpatient care (lack of time, multi-bed rooms, crisis orientation) hinder early and individualized implementation, a problem described in another study on palliative care in hospitals [49]. In contrast to previous research, respondents had no mistrust in the prognostic qualities of the tool, which would rule out lack of trust as a cause of failed implementation [42]. Nevertheless, the positive changes in the communication climate indicate potential for development. MINI may have the potential to support a shift towards a more open approach to dying and death, provided that structural barriers are actively addressed.

As our data suggests, MINI uptake tends to be higher when interventions are linked to specific courses of action and can be meaningfully integrated into existing processes, such as ward rounds or interdisciplinary meetings. The low uptake of SPICT and QPS highlights that inadequate guidance and uncertainty in use are significant barriers, a challenge also identified in advance care planning interventions, where patients often experience decision uncertainty due to complex information and a lack of clarity [50,51].

Our results highlight that organizational and hierarchical structures have a significant influence on who feels responsible for implementation, a circumstance that tends to hinder the active participation of nurses and residents. The crucial role of cross-team implementation is well documented and underlines that interprofessional collaboration increases the effectiveness of palliative care interventions [52]. Our results are consistent with this observation and suggest that cohesive teamwork may facilitate better integration of tools such as MINI [53]. The deviations from the original implementation of MINI, in particular the selective application of the SPICT criteria and the flexible use of intervention as a reflection tool, are common in practice, especially in complex clinical situations [54]. In comparison, however, it is evident that in addition to its function as a cognitive stimulus, MINI may also contribute to interprofessional communication processes and role perceptions. The intervention has been demonstrated to elicit individual reflection, whilst, under certain conditions, also promoting coordination within teams and across sectors. This aspect has received little attention in the literature to date. Despite the modest scale of this preliminary study, future research could explore confirmatory testing to further investigate these trends.

At the same time, similar hurdles to implementation are evident as in other studies on advanced care planning or identification tools in the last year of life [51]. This supports earlier criticism that complex interventions are often difficult to put into practice in clinical settings. In contrast to studies that attribute low adherence to a lack of acceptance [55], our study shows that the MINI was not successful due to organizational bottlenecks and unclear responsibilities despite general acceptance. In retrospect, we suspect that recruiting a person employed on the ward to the research team would have been a solution to the problem of a lack of responsibility. This would have ensured the presence of a constant reminder to the participants of the study. With regard to sustainability, our observation of the strong dependence on key persons is consistent with the results of numerous implementation studies [49]. While selective integration is successful, systematic anchoring in routines, e.g., through training, reminder systems or standard processes, remains a key challenge. In particular, the selective application of SPICT criteria and QPS reflects the findings that the scope and complexity of instruments are critical success factors for their long-term use.

A particular added value of this study lies in the differentiated analysis of occupational group-specific differences. Instead of examining physicians or nurses in isolation, the chosen approach enables a comparative perspective on both professional groups. The only moderate or even declining outcomes among non-physician staff, particularly with regard to legal issues such as living wills or powers of attorney, raise questions about the profession-sensitive design of such measures. The literature increasingly emphasizes that interprofessional interventions need to be developed and trained from multiple perspectives in order to be effective in heterogeneous teams [56], an aspect that is also supported by our results.

Moreover, the partial decline in the perceived responsibility of non-medical professionals for conversations about dying and death indicates a potential unintentional reinforcement of conventional role patterns. While some studies point to an increasing openness for end-of-life content [57], our data rather show a re-traditionalization in which physicians are again clearly perceived as the primary actors. This indicates that without explicit role clarification and structural support, interventions such as MINI could potentially reinforce existing hierarchies. This should be given greater consideration in future studies.

In summary, MINI appears to be a context-sensitive intervention that may help initiate conversations about prognosis and end-of-life care after minor adaptations. In this feasibility study, some indications were observed that the intervention supports prognostic thinking and stimulates interprofessional communication. These findings highlight the importance of structured, professionally sensitive implementation strategies and appropriate institutional framework conditions. Given the small sample size, feasibility design, and implementation challenges, results should be interpreted cautiously, and further research is needed to confirm effectiveness and generalizability.

**Limitations**

This study is limited by external challenges. The implementation of the intervention exposed a multitude of challenges. The COVID-19 pandemic led to a significant reduction in recruitment, as happened to numerous other research projects during the same period [58]. This interruption was due to factors such as lockdowns and the reallocation of resources. Also, the application of the intervention was not always consistent, and there were challenges in integrating it into everyday clinical practice, particularly due to the lack of clear guidance and structural barriers. The extent to which the intervention is implemented within teams depends on the dedication of individual key personnel. This commitment is reflected in the variations in progress observed among different professional groups.

This study was designed as a feasibility study, which inherently limits the generalizability of the findings due to the small sample size across all evaluation components. Moreover, reported improvements are based on self-reported data, which may be subject to social desirability bias or influenced by the participants’ subjective self-perceptions. All results must be viewed with caution due to the small sample size and the high loss of participants from the pre- to the post-survey. It is reasonable to assume that there is a bias in the post-survey, as it was particularly those HSCPs who were in favor of the intervention who took part. The actual values may therefore deviate from the values surveyed.

The findings are also subject to selection bias, as the interview results reflect the experiences and subjective assessments of ward staff who were directly involved in the implementation. Due to data protection regulations, we were not able to assess the accuracy of the MINI in identifying eligible patients, which limits the evaluation of implementation quality. However, it is important to emphasize that the aim of this study was not to assess the prognostic validity of the MINI, but rather to explore the feasibility of introducing a palliative care perspective on a curative ward. Furthermore, the study design did not include a validation of the healthcare professionals’ assessments trained in the use of MINI. As a result, staff had no feedback on the accuracy of their patient identification. A validation of these assessments by study personnel was not possible due to data protection constraints. Future studies should address these conditions in advance of implementation to allow for a more robust evaluation of both feasibility and prognostic validity.

However, variations in the effectiveness of the intervention across different professional groups suggest that further exploration is needed to understand why physicians benefited more than other medical staff. Additionally, the self-reported nature of these findings calls for further validation through objective assessments of participants’ actual competencies.

**Implications**

The findings suggest that the MINI represents a promising tool for raising awareness and facilitating communication about limited life expectancy, particularly when embedded within structural processes and supported by interprofessional communication. For long-term integration, complementary measures such as regular training, reflective practice opportunities, and stronger institutional support are essential.

Sustainable implementation of the intervention requires its stable and routine integration into daily clinical practice. This includes regular training sessions, structured team meetings, and the use of reminder systems. Long-term anchoring in routine care can only be achieved if institutional structures and clearly defined responsibilities actively support the use of interventions.

Future research should explore the specific effectiveness of individual components of the intervention across various care settings to better understand their respective contributions and contextual adaptability.

## 5. Conclusions

MINI may have the potential to encourage a more palliative-aware approach when treating seriously ill and dying patients. However, to realize its full potential and ensure long-term success, it is important to adapt the approach to a specific context, adjust the interventions as necessary, and integrate it thoughtfully and participatory within each setting.

## Figures and Tables

**Table 1:** Sociodemographic data of the participants.

Characteristic	Total Pre N = 34 (%)	Total Post N = 14(%)
Age		
Under 20 years	1 (2.9)	0 (0)
21–30 years	13 (38.2)	8 (57.1)
31–40 years	10 (29.4)	2 (14.3)
41–50 years	5 (14.7)	3 (21.4)
51–60 years	5 (14.7)	1 (7.1)
Gender		
Female	20 (58.8)	9 (64.3)
Male	14 (41.2)	5 (35.7)
Hospital ward		
Nephrology	18 (52.9)	9 (64.3)
Gynecology	16 (47.1)	5 (35.7)
Profession		
Physician	13 (38.2)	6 (42.9)
Nurse	17 (50.0)	6 (42.9)
Other ^1^	4 (11.8)	2 (14.2)
Work experience		
Under 1 year	5 (14.7)	3 (21.4)
1 to 5 years	9 (26.5)	5 (35.7)
6 to 10 years	4 (11.8)	1 (7.1)
11 to 20 years	11 (32.4)	4 (28.6)
More than 20 years	5 (14.7)	1 (7.1)
Experience in the treatment of seriously ill patients in the last year of life		
Under 1 year	6 (17.6)	4 (28.6)
1 to 5 years	9 (26.5)	6 (42.9)
6 to 10 years	9 (26.5)	0 (0)
11 to 20 years	6 (17.6)	3 (21.4)
More than 20 years	4 (11.8)	1 (7.1)
Additional qualification in palliative care		
Yes	4 (11.8)	0 (0)
No	30 (88.2)	14 (100)

^1^ Case management and psychologists.

**Table 2:** **Acceptability:** critical appraisal of implementation outcomes *, Proctor et al. 2011 [36].

Category	Critical Appraisal of Implementation Outcomes	SQ	SPICT	QPS
Acceptability	The MINI was well accepted, especially the SQ, which is seen as stimulating interprofessional exchange and reflection, while SPICT and QPS were predominantly rejected by physicians due to their complexity and lack of suitability for everyday use; the intervention promoted external cooperation, but showed ambivalent outcomes on internal communication within the team.	2 ***	0 **	0 **

* This scoring is inductively derived from the mixed-methods findings of this study. This scoring provides a rapid overview of the extent to which MINI was successfully implemented. ** 0: Not achieved; *** 2: Fully achieved.

**Table 3:** **Adoption:** critical appraisal of implementation outcomes *, Proctor et al. 2011 [36].

Category	Critical Appraisal of Implementation Outcomes	SQ	SPICT	QPS
Adoption	The adoption of the MINI was inconsistent and remained limited in routine practice. The SQ was used regularly and widely, leading to measurable increases in competence, particularly in the psychosocial area. SPICT and QPS, on the other hand, were rarely used due to unclear procedures and a lack of instructions and therefore had little outcome.	2 ***	0 **	0 **

* This scoring is inductively derived from the mixed-methods findings of this study. This scoring provides a rapid overview of the extent to which the MINI was successfully implemented. ** 0: Not achieved; *** 2: Fully achieved.

**Table 4:** Self-assessment scale with focus on responses of participants who agreed or partly agreed to the statement. Palliative Competence Test for Physicians (Palliative Kompetenztest (PKT)) [26].

I Think That I Am Capable	Pre n (%)			Post n (%)		
	Physicians Only	Other Medical Staff Only	Overall	Physicians Only	Other Medical Staff Only	Overall
to collect data that describe a patient’s pain in a differentiated way, n = 34/14.	11 (84.6)	19 (90.5)	30 (88.2)	5 (83.3)	6 (75.0)	11 (78.6)
to present the regional offers of specialized palliative care, n = 34/14. *p* = 0.020	5 (38.5)	8 (38.1)	13 (38.2)	2 (33.3)	5 (62.5)	7 (50.0)
to convince a colleague of the need for palliative support, n = 34/14.	12 (92.3)	19 (90.5)	31 (91.2)	6 (100)	7 (87.5)	13 (92.9)
recognize psychosocial problems and discuss them with patients and relatives, n = 34/14. *p* = 0.026	13 (100)	12 (57.1)	25 (73.5)	6 (100)	7 (87.5)	13 (92.9)
to organize a referral to hospice and palliative care services, n = 34/13.	7 (58.3)	12 (57.1)	19 (57.6)	5 (83.3)	4 (57.1)	9 (69.2)
to recognize the complex needs of a dying person and to react appropriately to them, n = 34/13.	9 (69.2)	16 (76.2)	25 (73.5)	6 (100)	7 (100)	13 (100)
to remain in conversation when the wish for euthanasia is expressed. n = 34/12.	9 (75.0)	12 (57.1)	21 (63.6)	5 (83.3)	5 (83.3)	10 (83.3)
to integrate cultural and spiritual aspects of dying and death into the treatment of seriously ill people, n = 34/12.	7 (53.8)	14 (66.7)	21 (61.8)	4 (66.7)	5 (83.3)	9 (75.0)
to recognize whether a person is suffering, even if the possibilities of communication are limited, n = 34/14.	12 (92.3)	19 (90.5)	31 (91.2)	5 (83.3)	8 (100)	13 (92.9)
to advise a patient on the framework of the living will and to draw one up with him/her, n = 34/12.	8 (61.5)	7 (33.3)	15 (44.1)	3 (50.0)	4 (66.7)	7 (58.3)

**Table 5:** Appropriateness: critical appraisal of implementation outcomes *, Proctor et al. 2011 [36].

Category	Critical Appraisal of Implementation Outcomes	SQ	SPICT	QPS
Appropriateness	While the conceptual relevance of MINI was broadly acknowledged, its practical appropriateness proved highly context-dependent. Successful application hinged on the constellation of who introduced the intervention, when, and in which setting. Physicians were generally attributed greater authority, particularly regarding prognostic communication, whereas nursing staff were seen as emotionally closer to patients. These role dynamics created ambiguity in responsibility and communication pathways. Moreover, variability in patient trajectories, especially outside oncology, posed challenges to the selective and meaningful application of MINI. The intervention’s perceived relevance was largely restricted to cancer care, limiting its broader applicability.	1 ***	0 **	0 **

* This scoring is inductively derived from the mixed-methods findings of this study. This scoring provides a rapid overview of the extent to which the MINI was successfully implemented. ** 0: Not achieved; *** 1: Partially achieved.

**Table 6:** Feasibility: critical appraisal of implementation outcomes *, Proctor et al. 2011 [36].

Category	Critical Appraisal of Implementation Outcomes	SQ	SPICT	QPS
Feasibility	The SQ was widely accepted as a low-threshold, time-efficient tool. In contrast, SPICT and the QPS were considered impractical due to time constraints, low applicability, and limited uptake, particularly in fast-paced inpatient settings. Structural challenges, including shift work, limited staff resources, and a lack of dedicated coordination mechanisms, further impeded interdisciplinary collaboration. Implementation often remained improvised and lacked systematic integration into clinical routines.	2 ***	0 **	0 **

* This scoring is inductively derived from the mixed-methods findings of this study. This scoring provides a rapid overview of the extent to which the MINI was successfully implemented. ** 0: Not achieved; *** 2: Fully achieved.

**Table 7:** Fidelity: critical appraisal of implementation outcomes *, Proctor et al. 2011 [36].

Category	Critical Appraisal of Implementation Outcomes	SQ	SPICT	QPS
Fidelity	Implementation fidelity was low. The MINI was rarely applied as designed but rather reduced to its most feasible components or adapted informally. SPICT, in particular, was frequently omitted. The COVID-19 pandemic and a months-long nursing strike severely disrupted implementation and training processes, contributing to fragmentation and inconsistencies. Without dedicated structures and prioritization within the clinical workflow, adherence to the original protocol remained limited.	1 ***	0 **	0 **

* This scoring is inductively derived from the mixed-methods findings of this study. This scoring provides a rapid overview of the extent to which the MINI was successfully implemented. ** 0: Not achieved; *** 1: Partially achieved.

**Table 8:** Implementation Costs: critical appraisal of implementation outcomes *, Proctor et al. 2011 [36].

Category	Critical Appraisal of Implementation Outcomes	SQ	SPICT	QPS
Implementation Costs	While financial costs were not a dominant issue, the intervention was highly sensitive to staff and time constraints. Without sustained investment in training, coordination, and protected time for patient engagement, MINI remained a marginal and non-prioritized addition to existing duties. Notably, time spent with patients either remained unchanged or increased, raising questions about added workload in the absence of systemic support.	n/a **	n/a **	n/a **

* This scoring is inductively derived from the mixed-methods findings of this study. This scoring provides a rapid overview to what extent the MINI was successfully implemented. ** n/a: not applicable.

**Table 9:** Penetration: critical appraisal of implementation outcomes *, Proctor et al. 2011 [36].

Category	Critical Appraisal of Implementation Outcomes	SQ	SPICT	QPS
Penetration	MINI was adopted selectively, mainly in oncology-related units and by senior medical staff. Broader dissemination was hampered by professional hierarchies, high workload, and low engagement from non-physician staff. The intervention failed to gain traction across diverse departments and patient groups, limiting its reach and systemic impact.	1 ***	0 **	0 **

* This scoring is inductively derived from the mixed-methods findings of this study. This scoring provides a rapid overview of the extent to which the MINI was successfully implemented. ** 0: Not achieved; *** 1: Partially achieved.

**Table 10:** Sustainability: critical appraisal of implementation outcomes *, Proctor et al. 2011 [36].

Category	Critical Appraisal of Implementation Outcomes	SQ	SPICT	QPS
Sustainability	While MINI generated reflective impulses, it failed to establish sustainable practice change. Its continued use was largely dependent on motivated individuals and external project support. In the absence of binding structures, ongoing training, and clearly defined responsibilities, long-term integration appeared unlikely. Prospects for cultural change remained aspirational rather than observable.	0 **	0 **	0 **

* This scoring is inductively derived from the mixed-methods findings of this study. This scoring provides a rapid overview of the extent to which the MINI was successfully implemented. ** 0: Not achieved.

## Data Availability

The authors will provide datasets generated and analyzed during the current study upon reasonably request.

## Abbreviations

The following abbreviations are used in this manuscript:

HSCP	Health and Social Care Practitioner
LYOL I/II	Last Year of Life (Part I and Part II)
MINI	Minimal Invasive Intervention
SQ	Surprise Question
SPICT^TM^	Supportive & Palliative Care Indicators Tool
